# Nocturnal heart rate variability in 1-year-old infants analyzed by using the Least Square Cosine Spectrum Method

**DOI:** 10.1186/s40101-017-0152-8

**Published:** 2017-09-16

**Authors:** Yuko Kochiya, Akari Hirabayashi, Yuhei Ichimaru

**Affiliations:** Tokyo Kasei University, 1-18-1 Kaga, Itabashi-ku, Tokyo, 173-8602 Japan

**Keywords:** Infant, Heart rate variability, Rhythm analysis, Nighttime, Least Square Cosine Spectrum Method

## Abstract

**Background:**

To evaluate the dynamic nature of nocturnal heart rate variability, RR intervals recorded with a wearable heart rate sensor were analyzed using the Least Square Cosine Spectrum Method.

**Methods:**

Six 1-year-old infants participated in the study. A wearable heart rate sensor was placed on their chest to measure RR intervals and 3-axis acceleration. Heartbeat time series were analyzed for every 30 s using the Least Square Cosine Spectrum Method, and an original parameter to quantify the regularity of respiratory-related heart rate rhythm was extracted and referred to as “RA (RA-COSPEC: Respiratory Area obtained by COSPEC).” The RA value is higher when a cosine curve is fitted to the original data series.

**Results:**

The time sequential changes of RA showed cyclic changes with significant rhythm during the night. The mean cycle length of RA was 70 ± 15 min, which is shorter than young adult’s cycle in our previous study. At the threshold level of RA greater than 3, the HR was significantly decreased compared with the RA value less than 3.

**Conclusions:**

The regularity of heart rate rhythm showed dynamic changes during the night in 1-year-old infants. Significant decrease of HR at the time of higher RA suggests the increase of parasympathetic activity. We suspect that the higher RA reflects the regular respiratory pattern during the night. This analysis system may be useful for quantitative assessment of regularity and dynamic changes of nocturnal heart rate variability in infants.

## Background

Polysomnography (PSG) is the objective standard method to evaluate the sleep pattern with high accuracy; it measures brain wave activity, eye movements, muscle activity, heart rate, and respiration during sleep. However, this procedure is time-consuming, requires instruments to monitor several physiological signals, needs specific skills, and is usually performed by specially trained technicians in restricted environments such as sleep laboratories. Therefore, its use in evaluating infants and young children is relatively limited. Several sleep studies have used subjective assessment tools for the evaluation of sleep conditions in children. These were performed using a questionnaire under free-living conditions. However, this method remains limited because child behavior must be observed continuously by the parent. Recently, a small, light-weight, wearable device has been developed to simultaneously record heart rate, 3-axis acceleration, and body temperature. This device can conveniently measure the RR interval, body position, and body movements under free-living conditions over 24 h. The beat-to-beat heart rate variability (HRV), determined from the RR intervals, is widely used as a noninvasive tool for assessing autonomic nervous function.

Autonomic nervous function changes are modified by sleep stages [1–4]. Thus, autonomic nervous function during sleep has recently been used to identify sleep stages in adults. However, the autonomic nervous system is immature in infants. They have a fast heart rate and rapid shallow breathing compared with adults. Although several studies have investigated the developmental changes of autonomic nervous function using HRV [5, 6], few studies have evaluated the dynamics of nocturnal changes of autonomic nervous function in infants.

With spectral analysis of HRV, the power spectrum contains two main frequency components: a low-frequency component (LF, 0.04–0.15 Hz) and a high-frequency component (HF, 0.15–0.50 Hz). The HF component is equivalent to respiratory sinus arrhythmia (RSA) and is mediated solely by parasympathetic activity [7]. RSA is one of the sinusoidal rhythms of the heartbeat in our pathophysiological system. It represents beat-to-beat fluctuations in heart rate associated with the respiratory cycle; the heart rate accelerates during inspiration and decelerates during expiration [8]. Fast Fourier transformation (FFT) is a powerful method to extract the spectral components of HRV and has been used in many studies that have investigated autonomic nervous system activity during sleep. This analysis method, however, requires interpolation to produce an evenly sampled time series, and the original data number is limited to be an integer power of 2. Then, analyses of HRV were usually performed at normal RR intervals of consecutive 5- or 10-min segments during the awake state, during stable NREM, and during REM sleep stages. In contrast, the Least Square Cosine Spectrum Method is useful to directly and quantitatively analyze the biological rhythm [9, 10]. This method has the advantage of being able to estimate the cosine components of data composed of unequal intervals and to arbitrarily configure the window range of analysis. This analysis method was applied with a window range of 30 s of heartbeat time series, which corresponded to PSG analysis epochs. In our previous study, we developed an original parameter to quantify the regularity of heart rate rhythm which reflects the respiratory pattern and referred to as “RA” [11]. We applied the Least Square Cosine Spectrum Method with a window range of 30 s of instantaneous heart rate in adults. The RA showed a 90-min rhythm during sleep, and the sensitivity and specificity for prediction of sleep stages 3 and 4 compared to PSG were 72.9 and 73.2%, respectively [12]. The purpose of the present study was to investigate the nocturnal heart rate variability in infants using the algorithm we developed. The previous 24-h period HR recording system was heavy, and it could therefore disturb infants’ sleep. In this study, heart rate and 3-axis acceleration were measured simultaneously using a wearable device.

## Methods

### Subjects

Six healthy infants (three females and three males), aged 12 months (range 11–13 months), participated in this study. All parents of the subjects were carefully instructed about the study, and all gave their written informed consent. The study was approved by the Tokyo-Kasei University Institutional Review Board.

Heart rate and 3-axis acceleration were continuously and simultaneously monitored using the wearable heart rate sensor “myBeat” (UNION TOOL CO., Tokyo, Japan). The device was placed on each subject’s left chest with disposable electrode (Vitrode. T-50, Nihon Kohden Co., Tokyo, Japan). Measurement was conducted at subjects’ homes under free-living conditions and started before bedtime and finished when the infants woke up in the morning. The parents were instructed to write down bedtime and waking times.

### Spectral analysis of HRV

The RR intervals and acceleration signals were recorded in the device memory and transferred to a computer for analysis. The instantaneous heart rate was calculated from the RR interval sequences. False-negative or false-positive RR intervals were excluded from the analysis. An automated computer analysis system was developed using Microsoft Visual Basic for Applications (VBA), and the Least Square Cosine Spectrum Method was applied with a window range of 30 s of the heartbeat time series. The formula of the cosine curve is as follows:

1 $$ Y=M+A\cos \left(2\pi t/\omega -\theta \right) $$

where *Y* is the estimated cosine curve, *M* is the MESOR (Midline Estimating Statistic Of Rhythm), *A* is amplitude (a measure of half the extent of predictable variation within a cycle), *t* is time, ω is period (duration of one cycle), and θ is acrophase (a measure of the time of overall high values recurring in each cycle).

By changing the period every 0.1 s sequentially, a high-frequency component of HRV was extracted. The cosine curve that showed the minimum value of the residual sum of squares calculated by subtracting the estimated cosine curve from the original data was referred to as the best-fit cosine curve. The series of the probability in each given cycle was calculated using the direct method by Sasaki [13, 14]. Then, the reciprocal logarithm of the probability was calculated and defined as the RA (RA-COSPEC: Respiratory Area obtained by COSPEC), which is expressed as:

2 $$ \mathrm{RA}=\log \left(1/\mathrm{Probability}\right) $$

The value of RA is higher when a cosine curve is fitted to the original data series.

In addition, we analyzed fitting curve for the time sequential changes of RA by using the Least Square Cosine Spectrum Method when the period was changing from 30 to 120 min. We calculated the probability of the fitness of cosine curve directly [13, 14].

### Analysis of triaxial acceleration

To determine the overall magnitude of physical activity, the vector magnitude (*G*) was calculated by taking the square root of the sum of squares from each axis:

3 $$ G=\sqrt{\left({X}^2+{Y}^2+{Z}^2\right)} $$

where *G* = 9.8 m/s^2^, the *X*-axis of triaxial acceleration reads right or left, the *Y*-axis reads up or down, and the *Z*-axis reads back or front movement. The sleeping position was estimated as in our previous study [15]: when the *Y*-axis value was more than −0.7, the subject was considered to be in the lying position. In addition, the left lateral position was defined as an *X*-axis value ≥ 0.5 G, the right lateral position was defined as an *X*-axis value < − 0.5 G, the supine position was defined as a *Z*-axis value ≥ 0.7 G, and the prone position was defined as a *Z*-axis value < − 0.7 G.

### Statistical analysis

The *t* test was used to examine the differences. *P* value < 0.05 was considered statistically significant.

## Results

### Heart rate and cosine curve for 30 s

**Fig. 1:**
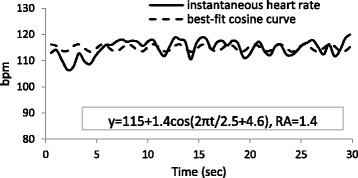
Instantaneous heart rate and best-fit cosine curve at 21:54 PM

**Fig. 2:**
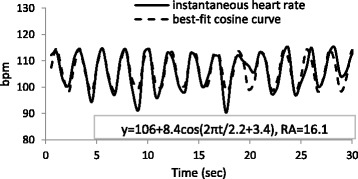
Instantaneous heart rate and best-fit cosine curve at 20:08 PM

Figures 1 and 2 show an original data series of the instantaneous heart rate and the results of the data calculated from the cosine fitting curve for 30 s. In Fig. 1, the period was 2.5 s, the MESOR was 115 bpm, and the amplitude was 1.4 bpm. In contrast, the frequency and amplitude of the heart beat changes are more regular in Fig. 2, which shows the period, MESOR, and amplitude of 2.2 s, 106 bpm, and 8.4 bpm, respectively. The RA, indicating fitness for the cosine curve, was higher in Fig. 2 than in Fig. 1. In the present study, these parameters were calculated for nocturnal data at 30-s intervals.

### Dynamic changes of heart rate variability and acceleration

**Fig. 3:**
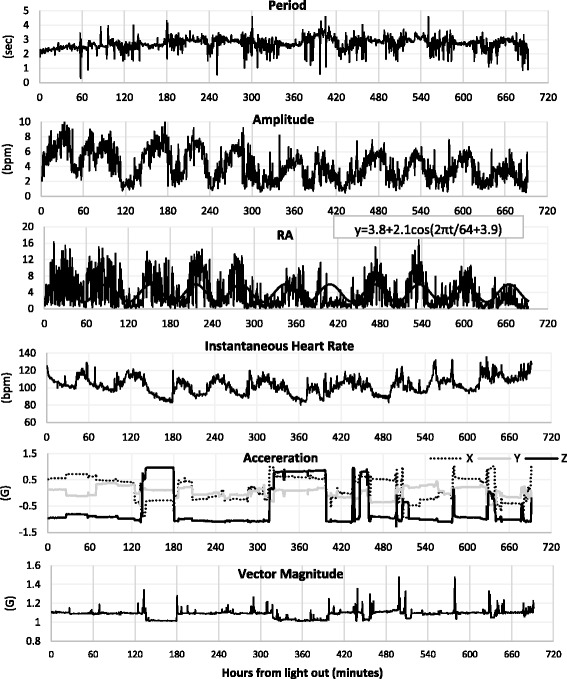
Changes of period, amplitude, RA, HR, 3-axis acceleration, and vector magnitude for a representative subject

Figure 3 illustrates the time sequential changes of period, amplitude, RA, mean value of instantaneous heart rate, 3-axis acceleration, and the vector magnitude calculated every 30 s in one representative subject. As shown by the values of the *X* and *Z*-axes, this subject spent more time in the prone position. The proportion of the supine position to time in bed was 6%, the prone position was 54%, the right lateral position was 3%, and the left lateral position was 36% in this subject. The vector magnitude increased mostly accompanied with position changes. The RA was characterized by 11 times repeated increases or decreases during the night (Fig. 3, 3rd row). The cyclic changes of RA were observed with a cycle length of 64 min, as measured by the Least Square Cosine Spectrum Method when the period was changing from 30 to 120 min. The mean HR was 103.0 ± 9.6 bpm (Fig. 3, in 4th row). The HR changed inversely with RA, that is, it showed a decrease at the time of higher RA, and it increased with lower RA. In this case, the mean HR was 98.8 ± 7.9 bpm when the RA value was greater than 3, but was 106.2 ± 9.6 bpm when was less than 3. This analysis was performed for all subjects, and the results are presented in Table 1. The HR was significantly lower when the RA was greater than 3 for all subjects.

**Table 1:** The comparison of mean HR when the RA was greater or less than the value 3

Subject	Mean HR when the RA value ≥ 3 (bpm)	Mean HR when the RA value < 3 (bpm)	*P* value
A	88.9 ± 5.6 (*n* = 273)	92.3 ± 9.0 (*n* = 1079)	< 0.001
B	104.4 ± 7.8 (*n* = 336)	114.4 ± 11.1 (*n* = 838)	< 0.001
C	105.8 ± 6.4 (*n* = 323)	115.1 ± 6.4 (*n* = 741)	< 0.001
D	89.3 ± 6.2 (*n* = 275)	94.3 ± 10.7 (*n* = 722)	< 0.001
E	102.6 ± 3.4 (*n* = 510)	109.1 ± 9.3 (*n* = 690)	< 0.001
F	98.8 ± 7.9 (*n* = 595)	106.2 ± 9.6 (*n* = 789)	< 0.001

We calculated the HR value when the RA was greater or less than 3. Values are expressed as mean ± SD. The difference of HR was assessed using the *t* test, and a *P* value < 0.05 was considered statistically significant. There was statistically significant difference in all subjects (*P* < 0.001)

**Fig. 4:**
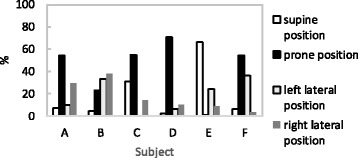
Individual proportions of body position to time in bed for six infants

For all subjects, mean time in bed by parental report was 606 ± 66 min. Figure 4 shows individual proportions of body position to time in bed. Among the six infants, four infants spent more than 50% of the time in bed in the prone position. The nocturnal individual profiles of RA are presented in Table 2. The mean RA was 2.8 ± 0.8. The cyclic changes of RA with significant rhythm were obtained from all subjects (*p* < 0.001), and the mean cycle length of RA was 70 ± 15 min.

**Table 2:** Individual nocturnal profiles of RA for six infants

Subject	Mean of RA	Max of RA	Cycle length of RA (min)
A	1.9	13.0	62
B	2.3	15.2	77
C	3.0	17.9	97
D	2.3	12.5	58
E	3.5	17.2	61
F	3.8	16.8	64
Average	2.8	15.4	70
SD	0.8	2.3	15

The RA value during the night averaged 2.8 ± 0.8 for the six subjects. The cyclic changes of RA were obtained from all subjects, and the mean cycle length of RA was 70 ± 15 min

### Assessment of RSA amplitude

The Least Square Cosine Spectrum Method is able to obtain the absolute value of amplitude, which approximates the original data if the estimated error of the cosine curve with the predicted period is smaller. The amplitude is used as an index of cardiac vagal activity. In this study, the mean values of period and amplitude were calculated when the probability as a function of cycle for a 30-s epoch was less than 0.001, that is, RA was greater than 3. The results for all subjects are shown in Table 3. The period and amplitude values averaged 2.8 ± 0.2 s and 4.2 ± 1.1 bpm for the six subjects, respectively. The amplitude differed among individuals; the range extended from 2.7 to 5.4 bpm.

**Table 3:** The period and amplitude of the cosine curve when the RA value was greater than 3

Subject	Period (sec)	Amplitude (bpm)
A (*n* = 273)	2.8	5.0
B (*n* = 336)	2.8	3.5
C (*n* = 323)	3.2	3.8
D (*n* = 275)	2.6	5.0
E (*n* = 510)	2.7	2.7
F (*n* = 595)	2.7	5.4
Average	2.8	4.2
SD	0.2	1.1

To evaluate the RSA amplitude used as an index of cardiac vagal activity, we calculated when the RA was greater than 3. The period and amplitude values averaged 2.8 ± 0.2 s and 4.2 ± 1.1 bpm for the six subjects, respectively. The amplitude differed among individuals; the range extended from 2.7 to 5.4 bpm

## Discussion

In this paper, we investigated the dynamic nature of nocturnal heart rate variability by using the Least Square Cosine Spectrum Method in 1-year-old infants and extracted a parameter referred to as “RA” to quantify the regularity of respiration-related heart rate rhythm. When heart rate time series fit well to cosine curve obtained from Least Square Cosine Spectrum Method, the RA value shown a higher value. This study demonstrated that the RA had repeated increases or decreases during the nighttime with the cycle length of 70 min. When setting the threshold level of RA at 3, a significant decrease of HR was observed compared with the threshold value of below 3, suggesting the increase of parasympathetic activity at this period.

The respiratory pattern shows dynamic changes during sleep. Respiration becomes regular during NREM sleep and irregular during REM sleep [16]. Bond et al. [17], who investigated subjects from 16 to 69 years old, reported that there was a relatively precise correlation between the respiratory pattern and rhythmic HRV, with a very uniform pattern of frequency and amplitude of sinus arrhythmia in deep sleep (NREM sleep stages 3 and 4). On the other hand, in REM sleep, there was complete dissociation of respiration and rhythmic heart rate. In our previous study comparing the RA and sleep stage by using PSG recordings in young adults, the RA was lower during REM sleep, MT (Movement Time), and awake and was higher during sleep stages 3 and 4 [12]. Consequently, we suspect that the higher RA obtained from in the present study reflects the regular respiratory pattern especially observed during deep sleep.

Concerning the period of cyclic changes of RA, the mean value in young adults was previously found to be 89 ± 11 min [11], while it was 70 ± 15 min in 1-year-old infants in the present study. Sleep alternates between REM and NREM sleep (akin to active and quiet sleep in infants). In young adults, REM sleep comprises about 20% of total sleep time, but in newborn infants, it comprises about 50% and decreases with age. The duration of the REM-NREM sleep cycle is generally thought to be about 90 min in young adults, but an infant’s cycle is shorter, lasting 40 to 60 min in newborn babies and infants, 60 to 80 min in 2- to 5-year-olds, and close to adult levels at 5 to 10 years [18]. A future study is required to clarify the possibility of the RA for estimating sleep cycle in infants.

Furthermore, another advantage of the Least Square Cosine Spectrum Method is that it is able to obtain the absolute value of the amplitude, which is used as an index of cardiac vagal activity. In this study, the mean amplitude of the cosine fitting curve was 4.2 ± 1.1 bpm when the RA was greater than 3. We should, in future, examine the useful method for evaluating the age-related changes of absolute amplitude in consideration of the differentiation of mean heart rate and respiratory rate between infants and adults. A limitation of this study is the small sample size, which was restricted to 1-year-old infants. Further studies including a larger sample are necessary to investigate developmental changes in autonomic nervous function. However, one should take into account that RSA amplitude is not only affected by vagal tone but also by respiratory frequency and tidal volume [19, 20].

Chronobiological studies have demonstrated that cardiovascular events occur frequently during night time or sleep time. Autonomic nervous function changes have been implicated in the development of cardiovascular disease. In addition, the association between infant sleeping position and risk of sudden infant death syndrome (SIDS) has been investigated. The method used in the present study could be used for the assessment of not only heart rate variability but also body position and body movement. Among the six infants, four infants spent more than 50% of the time in bed in the prone position. Hence, the application of the method to epidemiological studies investigating relationships between sleep position and autonomic nervous system function is expected.

## Conclusions

This study investigated the nocturnal heart rate variability in 1-year-old infants using the Least Square Cosine Spectrum Method. We extracted a parameter to quantify the regularity of respiration-related heart rate rhythm and referred to as “RA.” Our study indicates that (1) the RA showed cyclic changes with significant rhythm during the night, (2) the mean cycle length of RA was 70 min, and (3) the HR decreased at the time of higher RA, suggesting the increase of parasympathetic activity. We suspect that the higher RA reflects the regular respiratory pattern during the night. This analysis system may be useful for quantitative assessment of regularity and dynamic changes of nocturnal heart rate variability in infants.

## Abbreviations

FFT: Fast Fourier transformation
HF: High frequency
HRV: Heart rate variability
LF: Low frequency
PSG: Polysomnography
RSA: Respiratory sinus arrhythmia
SIDS: Sudden infant death syndrome
VBA: Visual Basic for Applications
